# Hand Pronation–Supination Movement as a Proxy for Remotely Monitoring Gait and Posture Stability in Parkinson’s Disease

**DOI:** 10.3390/s22051827

**Published:** 2022-02-25

**Authors:** Yusuf Ozgur Cakmak, Can Olcek, Burak Ozsoy, Prashanna Khwaounjoo, Gunes Kiziltan, Hulya Apaydin, Aysegul Günduz, Ozgur Oztop Cakmak, Sibel Ertan, Yasemin Gursoy-Ozdemir, Didem Gokcay

**Affiliations:** 1 Department of Anatomy, School of Biomedical Sciences, University of Otago, Dunedin 9054, New Zealand; prash.khwaounjoo@otago.ac.nz; 2 Medical Technologies Centre of Research Excellence, Auckland 1142, New Zealand; 3 Centre for Health Systems and Technology, Dunedin 9054, New Zealand; 4 Centre for Bioengineering and Nanotechnology, Point-of-Care Technologies, Dunedin 9054, New Zealand; 5 Department of Health Informatics, Middle East Technical University, Ankara 06800, Turkey; colcek@gmail.com (C.O.); didemgokcay@gmail.com (D.G.); 6 Global Dynamic Systems ARGE, Teknopark Istanbul, Istanbul 34906, Turkey; burakozsoy@gmail.com; 7 Department of Neurology, Cerrahpasa Medical Faculty, Istanbul University-Cerrahpasa, Istanbul 34098, Turkey; guneskiziltan@gmail.com (G.K.); apaydin.hulya@hotmail.com (H.A.); aysegul.gunduz@iuc.edu.tr (A.G.); 8 Department of Neurology, School of Medicine, Koç University, Istanbul 34010, Turkey; ooztop@ku.edu.tr (O.O.C.); sertan@ku.edu.tr (S.E.); ygursoy@ku.edu.tr (Y.G.-O.); 9 Research Centre for Translational Medicine (KUTTAM), Koç University, Istanbul 34010, Turkey

**Keywords:** gait, posture, Parkinson’s disease, pronation, supination, bulbar anomalies

## Abstract

The Unified Parkinson’s Disease Rating Scale (UPDRS) is a subjective Parkinson’s Disease (PD) physician scoring/monitoring system. To date, there is no single upper limb wearable/non-contact system that can be used objectively to assess all UPDRS-III motor system subgroups (i.e., tremor (T), rigidity (R), bradykinesia (B), gait and posture (GP), and bulbar anomalies (BA)). We evaluated the use of a non-contact hand motion tracking system for potential extraction of GP information using forearm pronation–supination (P/S) motion parameters (speed, acceleration, and frequency). Twenty-four patients with idiopathic PD participated, and their UPDRS data were recorded bilaterally by physicians. Pearson’s correlation, regression analyses, and Monte Carlo validation was conducted for all combinations of UPDRS subgroups versus motion parameters. In the 262,125 regression models that were trained and tested, the models within 1% of the lowest error showed that the frequency of P/S contributes to approximately one third of all models; while speed and acceleration also contribute significantly to the prediction of GP from the left-hand motion of right handed patients. In short, the P/S better indicated GP when performed with the non-dominant hand. There was also a significant negative correlation (with medium to large effect size, range: 0.3–0.58) between the P/S speed and the single BA score for both forearms and combined UPDRS score for the dominant hand. This study highlights the potential use of wearable or non-contact systems for forearm P/S to remotely monitor and predict the GP information in PD.

## 1. Introduction

Parkinson’s disease (PD) is a neurodegenerative disorder characterized by both motor and non-motor symptoms. Symptoms of motor dysfunction comprise tremor (T), rigidity—stiffness (R), bradykinesia—slowness in movement (B), gait—postural instability (GP), and bulbar abnormalities that include difficulties with speech and facial expressions (BA). Symptoms of non-motor dysfunction, such as pain, saliva control, sleeping, etc., are out of the scope of this study. In Parkinson’s disease, the Unified Parkinson’s Disease Rating Scale (UPDRS) is the most common tool used in motor symptom assessment. Researchers have previously studied rhythmic pronation and supination mechanics [1,2,3], but UPDRS motor scaling does not consider pronation–supination to be a proxy for gait ability; it is only used for assessment of bradykinesia/rigidity. Additionally, no study has examined the possible relationship between forearm pronation–supination and gait/position instability in Parkinson’s disease. A variety of biomedical wrist wearable devices (e.g., smartwatches, accelerometers, etc.) exist for monitoring motor symptoms in Parkinson’s patients. There is, however, no wrist wearable or non-contact wrist motion monitoring system that can be utilized for objectively assessing all five UPDRS III motor symptoms (T, R, B, GP, BA), by monitoring Parkinson’s disease motor symptoms. At present, wrist wearables can provide T, B, and R information, but not GP or BA. The main limitation of these devices is not their functional capabilities, but rather the lack of knowledge of wrist movements that can be associated with GP and BA.

Pronation and supination of the forearm are among the most complicated movements that primates can perform. During pronation, most quadrupedal primates exhibit a similarly extended wrist morphology [4]. The mechanisms underlying these movements are based on the circular head of the radius bone swiveling over the ulna during supination and becoming parallel to the ulna. Furthermore, the literature indicates that the erect postures of quadrupedal animals are associated with their circular heads of the radius bone, thus with their ability to pronate [5,6,7,8,9,10,11,12,13]. Moreover, it has been suggested that amphibious animals, such as lizards and crocodiles, cannot pronate their forearms because they lack a circular radial head and therefore cannot become erect on the forearms while moving on land. On the other hand, land mammals, such as cats and most primates (including humans), with circular radial heads, can actively pronate their forearms during quadrupedal locomotion [5,6,7,8,9,10,11,12,13].

The ability to supinate is also reported to be positively correlated with the mass of quadrupedal mammals (e.g., bears supinate more than cheetahs) [14]. This indicates that the pronation and supination abilities of the forearm are advantageous for gait and posture stability in quadrupedal locomotion. In this context, gait disorders may benefit from an understanding of pronation and supination movements of the forearm in quadrupedal locomotion, as well as vestiges from the evolution of bipedal movement in humans, especially for development of gait monitoring applications.

It has also been investigated whether bipedal mammals, including humans, use quadrupedal coordination in order to control their gait, based on central pattern generators (CPGs) [15,16]. Interlimb coordination between human upper and lower limbs has been found to be similar to that of quadrupedals [15,16,17]. It has also been demonstrated that the upper limb displays similar characteristics to the hind limb only during rhythmic movements [15,16,18,19]. In this context, it has been postulated that the neuronal coordination of the CPGs that modulate the upper and lower limbs is conserved in humans [15,16]. Another mechanism of CPG is the reciprocal inhibition of two neurons in order to produce alternate activity patterns [19,20]. Finally, a recent multi-array cortical recording study in humans has demonstrated that neurons in the hand knob area of the premotor cortex are not specific to hand movements but are involved in the movement of all four limbs. It was also demonstrated that the neural code was associated with the matching movements of all four limbs [21].

In light of the beneficial effects of greater pronation–supination on gait and postural stability in mammals to conserved interlimb coordination via CPGs, as well as reports on multi-array cortical recordings from the human premotor cortex that showed the integration of all four limb movements, we hypothesize that the CPG controlling gait function might be related to the motor circuit generating sequential rhythmic pronation-supination of the forearm.

In the current practice, there is no unilaterally worn device or wearable device (e.g., smartwatch) that can collect or provide gait and bulbar anomaly information that are the main components of the UPDRS. Further, in order for wearable devices or mobile phone-based applications to be able to provide gait information in Parkinson’s disease, walking or sit-to-stand tasks are necessary. Such tasks are also associated with the risk of falling in PD. Traditionally, pronation and supination movements of the wrist are considered as a part of the UPDRS motor scoring for bradykinesia. Pronation and supination have not been utilized as an indirect measure to obtain information about the gait and/or bulbar anomalies of the PD. To our knowledge, this is a pioneering study, which aims to investigate the potential use of wrist pronation–supination patterns to provide gait and/or bulbar abnormality information associated with PD. This study opens up the possibility to use single wrist wearables (e.g., smartwatches) for remote monitoring and reporting of PD motor symptoms associated with gait and bulbar anomalies without conducting a walking or sit-to-stand task.

## 2. Materials and Methods

### 2.1. Research Participants

In a cross-sectional study, twenty-four patients with idiopathic PD (17 men, 7 women; mean age ± SD = 57.08 ± 8.91 years) participated. Each subject provided written informed consent. It was approved by the Ethics Committee of Koç University, Turkey (Approval Number: 2015.091.IRB1.018) and conducted according to the Declaration of Helsinki Ethical Principles for Medical Research Involving Humans. The wrist P/S motion capture data were collected during a two-armed clinical trial (first arm: two sessions for ten patients, second arm: three sessions for fourteen patients).

All patients were diagnosed with PD by a neurologist who was an expert in movement disorders, using the UK Parkinson’s Disease Society Brain Bank clinical diagnostic criteria [22]. All patients underwent a detailed neurological examination, and Parkinsonian features were rated according to Hoehn and Yahr Scale (Table 1). Furthermore, all patients used dopaminergic replacement treatment (disease duration ± SD = 8.04 ± 3.88 years) but were asked to stop taking their medications 12-h before the recording sessions. Patients were also evaluated for hand dominance: 23 were right-handed, only one patient was left-handed. Patients with a disease duration of longer than a year or a Hoehn and Yahr stage ≥2 were included. Patients with cognitive impairment that might prevent cooperation during testing and patients with other neurological or systemic diseases were excluded at this stage.

### 2.2. Assessing Forearm Pronation–Supination and Collection of the UPDRS Scores

The UPDRS has numerous subsections to assess mood, activities of daily living, motor symptoms examinations, and complications of therapy and is routinely used in daily clinical practice for PD assessment. However, only motor symptoms are of concern for this study.

The purpose of UPDRS Part III is to examine motor symptoms. The UPDRS part III assesses symptoms on a scale of 0 to 4 (0 being no symptom, 4 being the most severe). UPDRS Part-III sub-scores are classified and analyzed in terms of tremor (at rest and action/postural tremor), rigidity (judged on passive movements of major joints), bradykinesia (finger taps, opening/closing hands rapidly, rapid alternating movements of the hands-pronation-supination, leg agility, body bradykinesia or hypokinesia), gait and postural instability (arising from the chair, posture, gait based on walking, postural stability), and bulbar anomalies (speech and facial expression), as previously described [23,24].

PD has a prevalent unilateral insurgence. Accordingly, UPDRS part III has lateralized subitems for tremor, rigidity, and bradykinesia, and the traditional approach involves considering the dominant side symptom scores for UPDRS part III motor scores. The present study collected scores from both extremities (with dominant and nondominant symptoms) to conduct correlation analyses with motion parameters obtained from a digital device, on each extremity. UPDRS part III assessment of both extremities is a novel method to investigate subtle differences in the UPDRS III scores on both extremities separately, since clinical practice typically includes only scores for the extremity with the dominant symptoms. Considering that UPDRS scores are determined by subjective judgments of the clinicians, we collected scores from two neurologists to maintain validity.

Two neurologists evaluated the patients’ motor symptoms using the UPDRS Part III scores bilaterally before collecting non-contact hand tracking data using an infrared camera. An infrared hand tracking camera (Leap Motion, Inc., San Francisco, CA, USA) and a consumer-level laptop were used to record data. In this investigation, patients were seated facing the back of a laptop without being able to see the recording display on the laptop screen. At first, patients were asked to place their hands above the controller to test the non-contact system’s ability to capture their hands’ position. Pronation–supination (P/S) movement is described to the patients by the same neurologists who were involved in UPDRS part III bradykinesia scoring, including the same P/S movement-based scoring.

The averages obtained from the two neurologists and the right and left sides were taken as the UPDRS III scores (which were initially $\mu_{left}\pm SD_{left}=11.49\;\pm 4.61\;\mathrm{and}\;\mu_{right}\pm SD_{right}=12.28\;\pm 5.15$). In our study, patients were asked to come for multiple visits and to participate in P/S movement recording sessions after at least one week. During the two-armed clinical trial, in which wrist P/S motion capture data were collected, 14 patients visited the hospital three times, and 10 visited twice.

To keep the dataset homogeneous for right-hand preference and right-hand dominant symptoms, one patient with left-hand preference and three patients with left-hand symptom dominancy were excluded. Each recording session was completed in six successive parts. More specifically, pronation–supination movements were repeated three times for both hands, in alternate order, and the movements incorporated at least three complete cycles of P/S, independently from the duration. Unfortunately, several patients were unable to complete the whole exercise. Thus, some PD patients had 18 movement records, while others had fewer. The final set of data included 274 distinct pronation–supination records from all patients (i.e., approximately 13 movement records per patient).

### 2.3. Measurement Device

Leap Motion (Leap Motion, Inc., San Francisco, CA, USA) is a popular low-cost gaming controller that captures hand and finger gestures. In spite of its compact size, it has infrared cameras that capture stereo images in grayscale. As opposed to similar devices that utilize depth maps, Leap Motion tracks movement using advanced algorithms [25] embedded in its standard development kit (SDK).

The controller samples the distances of finger joints at approximately 100 Hz. Furthermore, a recent analysis was done by Weichert et al. [26] and revealed that the average accuracy of these measurements (0.7 mm in all three axes) is close to that of the finest movements of the human hand (0.4 mm).

### 2.4. Motor Tasks and Extracted Features

**Figure 1:**
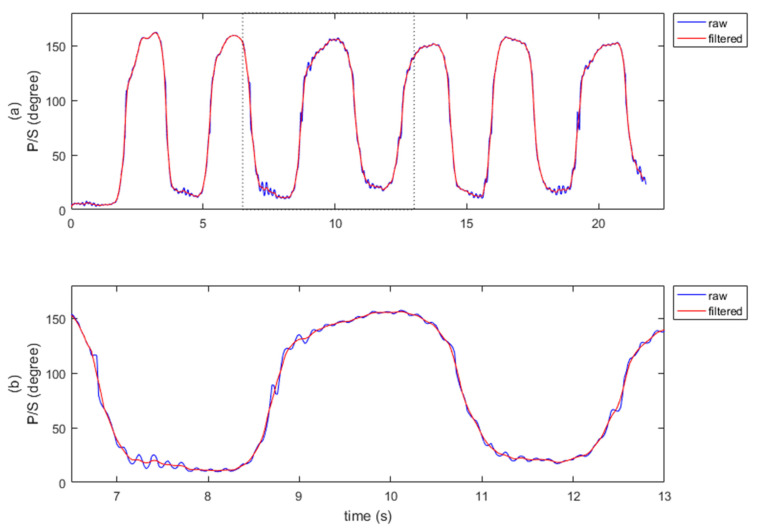
Application of the moving average filter. (**a**) The original raw signal and the resulting waveform after filtering. (**b**) Enlarged signal bounded by the dashed lines in part (**a**) to observe the details of the tremor.

All patients’ P/S raw gesture data were recorded using custom software developed using Leap Motion SDK. During P/S sessions, participants are observed by the same neurologist who scored their UPDRS scores. The raw data were processed in order to extract features that defined the characteristics of patient motions. The rotations of the wrist were processed on a single axis in terms of degrees. A P/S task involves rapid, repetitive movements around the roll axis consisting of multiple local minimal and maximal values (Video S1). The maximum and minimum were extracted as features by tracking the local minimum/maximum in the motion sequence and identifying the last qualifying data point before the movement reversed its direction. The consecutive markers were also used to calculate three final features: speed, acceleration, and frequency. These three features have also been examined in previous studies [27,28] and have been shown to be useful for assessing bradykinesia. In this study, we extended the application of these parameters to all compartments of the UPDRS. Figure 1 shows a sample raw dataset collected using this software and methodology.

## 3. Correlation Analysis of UPDRS Scores and Pronation–Supination Motion Parameters

### 3.1. Data Preprocessing

Occasional glitches and noise in the data originating from the initiation or termination of movements were excluded from all records. The exclusion procedure was conducted by visually inspecting and manually marking invalid sections by a single investigator (second author, CO). A detailed explanation of these exclusion processes can be found in Supplementary Figure S5.

**Figure 2:**
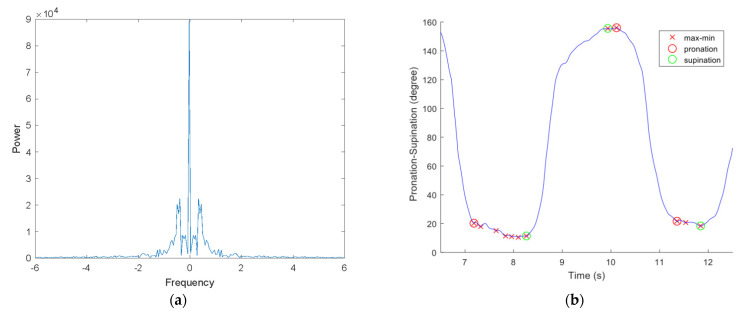
Important features of the P/S movement signal after preprocessing. (**a**) Frequency (Hertz) spectrum and (**b**) maximum and minimum points during pronation–supination (P/S) movement (“×” marks indicate the extrema points found by the marking process, circles indicate retained minimums and maximums after discarding non-consecutive extrema during P or S movement).

Tremors that occurred during data collection appeared as oscillations that caused inaccuracies in the detected minima and maxima. Waveforms of the motions were analyzed using frequency spectra in order to resolve this problem. A typical spectrum contains a significant response below 5 Hz (Figure 2a). Based on the sampling rate, a moving average filter with a window size of seven was applied to smooth the signal, as shown in Figure 1. In saddle points, where the motion reached extremes, several patients had double peaks, which was expected due to tremor. Considering that our feature selection was based on differences between the length of time and the angle of a particular movement, we were able to use consecutive minima and maxima pairs to calculate pronation and supination features. As seen in Figure 2b, only consecutive minima and maxima were marked for further feature extraction. As explained in the analysis section, we used averaged features derived from several minima and maxima pairs. For these analyses, movement consistency was an important attribute. Therefore, we included only signals that were long enough to demonstrate four consecutive minimum–maximum pairs in a single pronation and supination motion. Signals containing fewer than four minimum-maximum points were excluded.

In total, 77 of the 274 recordings were excluded due to erroneous/incomplete pronation–supination data where the patient had performed additional or incomplete movements. Therefore, only 197 of the 274 recordings passed the quality control steps. The data analysis algorithm chart is provided in Supplementary Figure S5.

### 3.2. Analyses

Following data preprocessing, the remaining datasets (197) were used for feature extraction. In this phase, three specific features, $f_{1},\;f_{2},$ and $f_{3}$ that stand for speed, acceleration, and frequency were calculated, starting with the first minima marker. Means of the calculated values for each consecutive minima-maxima point (Equations (1) and (2)) in a single record were accepted in the final feature metric (Equations (3)–(5)). Marked extrema points enabled separate metrics for pronation and supination phases. Furthermore, wrist features for combined motion, without separating the pronation and supination components, were also computed, as shown in Equations (6)–(8) below:

(1) $$d\emptyset =\left|\emptyset_{min}-\emptyset_{max}\right|$$

(2) $$dt=\left|t_{min}-t_{max}\right|$$

(3) $$f_{1}=\frac{1}{n}\sum d\emptyset /dt$$

(4) $$f_{2}=\frac{1}{n}\sum d\emptyset /dt^{2}$$

(5) $$f_{3}=\frac{1}{n}\sum 1/dt$$

(6) $$f_{1}^{wrist}=\frac{1}{n}\sum (d\emptyset_{pro}+d\emptyset_{sup})/\left(dt_{pro}+dt_{sup}\right)$$

(7) $$f_{2}^{wrist}=\frac{1}{n}\sum (d\emptyset_{pro}+d\emptyset_{sup})/{\left(dt_{pro}+dt_{sup}\right)}^{2}$$

(8) $$f_{3}^{wrist}=\frac{1}{n}\sum 1/\left(dt_{pro}+dt_{sup}\right)$$

where ∅ = wrist angle, $\emptyset_{min}$ = angle at local minima, $\emptyset_{max}$ = angle at local maxima, *t* = time, $t_{min}$ = time at local minima, $t_{max}$ = time at local maxima, n = number of valid consecutive extrema points.

As shown in Formulas (6)–(8) the pronation and supination movements are treated as a single movement, regardless of the pause between them. Pronation and supination angles were added together, meaning that wrist movements from one direction to another were not considered in the analysis since the entire pronation and supination movement was considered as continuous movement.

In the final dataset, Pearson’s correlations between UPDRS scores and the aforementioned motion features were calculated. In addition, since UPDRS part III is comprised of five subgroups/scores (i.e., T, R, B, GP, BA), correlation analyses have been performed for each UPDRS part III total score, as well as for each individual subgroup and combinations of subgroups (i.e., T + R, T + B, T + BA, etc.) for a total of 18 combined UPDRS scores. A key purpose of this method was to explore and highlight patterns in the relationships between subgroups.

### 3.3. Predictive Analyses of Gait and Postural Instability

In addition to correlation analyses, further analysis of GP UPDRS scores was conducted to determine if there was an association between the GP scores and upper limb pronation–supination motion. First, we tested whether GP UPDRS scores from different subgroup combinations were significantly correlated with P/S motion features. In order to understand how features extracted from a given task contribute to GP scores, multiple linear regression models were constructed using different feature combinations. In these models, we examined whether GP could be predicted using a multiplicity of features from pronation and supination motion data. The regression models were extensive: initial models included any two different motion features as independent variables, then another set of models comprised all possible combinations of three, four, and n features (*n* = 18 as seen in supplementary Figures S1–S4). All possible combinations of multiple feature subsets ($n_{models}=\text{262,125}$) were used to create and test these models. In order to perform further regression analyses, models with minimal root mean square error (RMSE) were selected from within 0.1% ($n_{models}\cdot 0.001=262$) of all models. Monte Carlo cross-validation was applied to these models, which were thus trained and tested in three different train versus test cases. The first case contained 90%, the second test contained 75%, and the third case contained 50% of the data for training while the remaining data were used during testing. All training sets were trained 1000 times, wherein the training sets were randomly sampled, and the remaining data were used to test the model’s accuracy. The RMSE between the actual and predicted GP scores were reported for the test data. In addition, the training errors were also reported in terms of RMSE for the training set. These data were aggregated for all of the 1000 random training iterations by computing the mean for each of the three cases. The selected models indicated that the lowest RMSE values were achieved by models that used only a few features.

### 3.4. Principal Component Analysis (PCA)

In the context of the linear regression models, not all of the features contribute similarly to the solution. The feature space can be further reduced by pruning features that do not play an important role in GP prediction. We ran PCA on the dataset containing all 18 features in order to understand the features with the biggest impact. PCA is a useful technique to reduce a higher-dimensional representation into a lower-dimensional set by joining related components together. For this purpose, a covariance matrix is generated from the features of multiple subjects. Then, eigenvalues are generated for the features that are bundled together, showing the amount of their impact on the spread of the entire dataset. Based on the largest eigenvalues, corresponding eigenvectors that indicate the combined features are generated. After eliminating the eigenvectors that correspond to the insignificant eigenvalues that do not have an important contribution to the variance in the dataset, the percentage of variance can be computed in the new feature space. It is a desired property of PCA to keep as much of the variance in the original dataset as the variance of the new feature space.

### 3.5. Statistical Analyses

All statistical analyses were performed using the MATLAB (MathWorks R2016a) Statistics and Machine Learning Toolbox. Signal processing phases were also developed in MATLAB (MathWorks R2016a) using the Fourier Analysis and Filtering functions. A multiple comparisons correction was required to determine the statistical significance of the linear regression models due to the differences in the number of correlated features used in each model (i.e., each prediction was correlated to various numbers of features originating from the same set of measurements). Therefore, a Bonferroni correction [29] was applied.

### 3.6. Results

Our assessments yielded measurements of speed, acceleration, and frequency, referred to as $f_{1},\;f_{2},$ and $f_{3}$, respectively. Initially, to investigate the relationships, we correlated UPDRS subgroups with the features extracted from the pronation–supination data using Pearson’s correlation score, *r*. In the presentation below, large effect size stands for *r* ≥ 0.5, small effect size stands for *r* < 0.3 and medium effect size means 0.3 ≤r<0.5, where r is the Pearson’s correlation coefficient.

**Figure 3:**
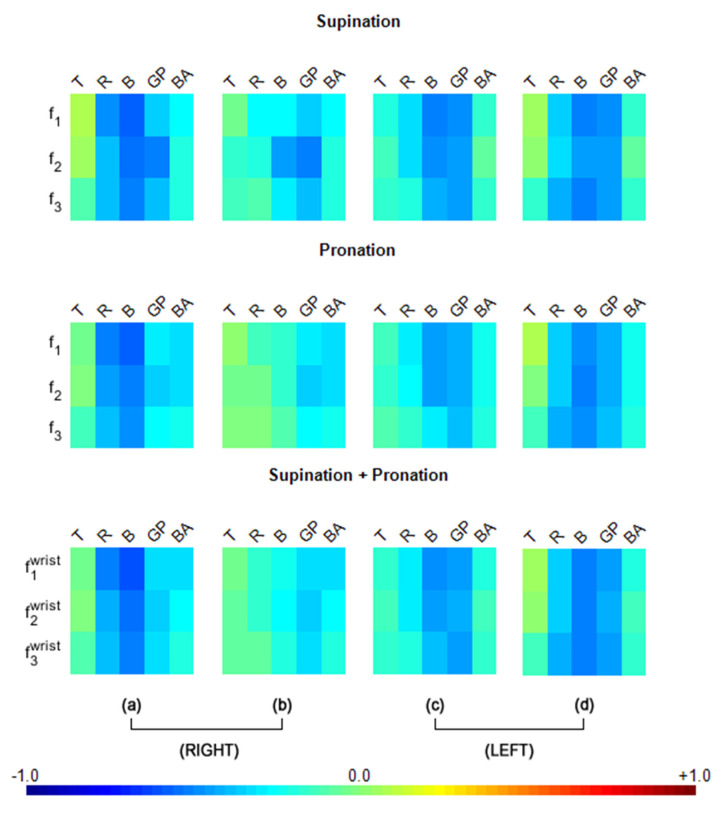
Pearson’s correlations between the extracted digital motion features from the right hand (**a**,**b**) and left hand (**c**,**d**) with the UPDRS scores from the right side (**a**,**d**), left side (**b**,**c**) and center (only for the GP score). Rows: features (*f*_1_: speed, *f*_2_: acceleration, *f*_3_: frequency); columns: UPDRS scores (T = tremor, R = rigidity, B = bradykinesia, GP = gait and postural Instability, BA = bulbar anomalies). Values above 0.2 and below −0.2 were significant at the *p* < 0.05 level (Bonferroni corrected).

As seen in Figure 3a, for the right UPDRS data, the $f_{1}$ (speed) and bradykinesia (B) were strongly correlated ($r_{1\left(B\right)}^{sup}=-0.60,\;r_{1\left(B\right)}^{pro}=-0.60,\;r_{1\left(B\right)}^{wrist}=-0.62$), with a large effect size) for both the pronation and supination components of right-hand motion. Furthermore, the $f_{1}$ for rigidity (R) followed the bradykinesia results with a similar effect size ($r_{1\left(R\right)}^{sup}=-0.50,\;r_{1\left(R\right)}^{pro}=-0.52,\;r_{1\left(R\right)}^{wrist}=-0.53$). Additionally, gait and postural instability (GP) were strongly correlated to $f_{2}$ (acceleration) for the supination component ($r_{2\left(GP\right)}^{sup}=-0.51$) also with large effect size, although GP is evaluated centrally, not ipsilaterally in UPDRS.

Although the other features ($f_{1},\;f_{3}$) were not as strongly correlated as the $f_{2}$ results in GP of Figure 3a, they were correlated with medium effect sizes. The combinations of UPDRS subgroup scores were also meaningful. To explore the entire set of combinations, we added individual UPDRS subgroup scores in sets of 2, 3, 4 and 5. As seen in Supplementary Figure S1, R + B was most strongly correlated ($r_{1\left(R+B\right)}^{sup}=-0.62,\;r_{1\left(R+B\right)}^{pro}=-0.63,\;r_{1\left(R+B\right)}^{wrist}=-0.65$) with the features, with large effect sizes. As expected, R + B was not only the most strongly correlated combination in the pronation–supination movements, but it also improved the individual UPDRS score subgroup correlations.

Among all combinations of UPDRS scores, those with GP stood out as the third most highly correlated component, with strong R and B correlations to the motion features. Correlations between features $f_{1}-f_{3}$ and GP warrants a closer inspection because, unlike R and B, which are naturally expected to correlate with motion speed, acceleration, and frequency, a relationship between GP and P/S has not yet been reported. While the correlation values of R + GP in $f_{2}$ ($r_{2\left(R+GP\right)}^{sup}=-0.55$) and R + B + GP in $f_{1}$ ($r_{1\left(R+B+GP\right)}^{sup}=-0.61$) were close to R + B for supination, pronation had similar R + B + GP ($r_{1\left(R+B+GP\right)}^{pro}=-0.60$) and R + B + GP + BA ($r_{1\left(R+B+GP+BA\right)}^{pro}=-0.58$) correlation values. Furthermore, combined $f_{1}$(speed) had slightly higher *r* values for B + GP ($r_{1\left(B+GP\right)}^{wrist}=-0.58$), R + B + GP ($r_{1\left(R+B+GP\right)}^{wrist}=-0.62$), and R + B + GP + BA ($r_{1\left(R+B+GP+BA\right)}^{wrist}=-0.60$). Correlations with the entire set of combinations is provided in Supplementary Figure S1.

Left-hand motion correlations with ipsilateral UPDRS scores (i.e., UPDRS scores from the left side) revealed high values, especially in the B and GP measurements, as shown in Figure 3c. While almost all of the combined UPDRS scores were strongly correlated as shown in Supplementary Figure S2, the correlations of the motion features with GP were stronger than those for the right hand (Figure 3a). When GP correlations were considered, a medium effect size (0.3 ≤r<0.5) was observable in every case for Figure 3c. According to our correlation analysis, GP was consistently found to be an important contributor to many of the moderate and strong correlations within all three motion features, despite being measured centrally.

When the right-hand motion data were analyzed for correlations with contralateral (i.e., left side) UPDRS scores (Figure 3b), very low effect sizes (r<0.3) were revealed for T, R, B, and BA but not for GP. In terms of UPDRS combinations, the combinations that included GP, such as B + GP, GP + BA and R + GP + BA scored moderate to high correlations for *f*_1_ (speed) or $f_{2}$ (acceleration). Few correlations had large effect sizes ($r_{2\left(GP\right)}^{sup}=-0.51,\;r_{2\left(R+GP\right)}^{sup}=-0.52,\;r_{2\left(B+GP\right)}^{sup}=-0.54$), as observed in Supplementary Figure S3.

Left-hand motion correlations with contralateral UPDRS scores (i.e., UPDRS scores from the right side) revealed high values, especially in the B and GP measurements, as seen in Figure 3d. While almost all of the combined UPDRS scores were strongly correlated (as shown in Supplementary Figure S4), correlations of the motion features with GP(Figure 3d) were stronger than those for the right hand (Figure 3b). When GP correlations were considered, a medium effect size (0.3 ≤r<0.5) was observable in every case (Figure 3d).

This correlation study indicated that although measured centrally, GP stood out as a consistent player in many of the moderate and strong correlations across all three motion features of the right and left hands. However, the correlations of GP with the non-dominant hand motion features (i.e., the left-hand features) were higher than those of the dominant hand (i.e., the right hand).While correlations of the UPDRS sub-group bradykinesia (B) are also observed to be high ipsilaterally for the right-hand motion features as well as ipsilaterally and contralaterally for the left-hand motion features, this characteristic has been established in the literature and it can be expected since the UPDRS is based on the sides of the body and innervation of the face is also innervated bilaterally. As such, Figure 3 provides important insights, predominantly for GP correlations.

### 3.7. Linear Regression Model

**Figure 4:**
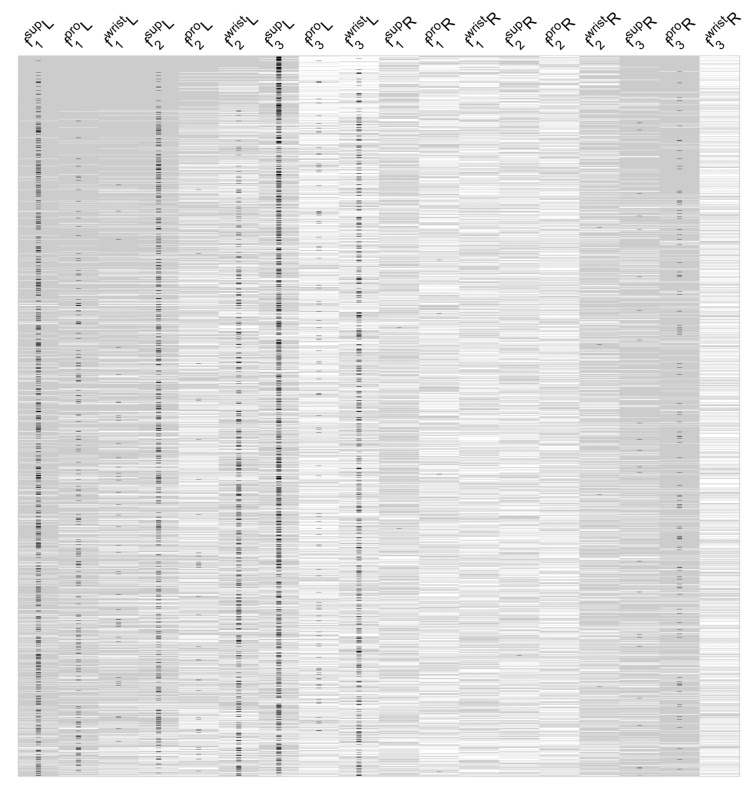
List of the features in top 1% (2621) of the regression models with lowest RMSE, ordered with respect to increasing RMSE (range: 1.20–1.24) (rows: different regression models, columns: model features; L = left hand, R = right hand from which features were extracted; white indicates absent feature, gray indicates contributing feature, black marks the significant features; *f*_1_, *f*_2_, *f*_3_ are speed, acceleration and frequency).

There were numerous, significant high correlations between the GP scores and the assessed motion features, indicating that a linear regression model could be used to predict GP UPDRS using these motion features. Among all the multiple linear regression models used for predicting GP scores, the models exhibiting the lowest RMSEs were identified. We employed the 1% of 262,125 models with the lowest RMSE values ($n_{models}*0.01=2621$) for this purpose. Figure 4 shows the significance of the features used in these models, highlighting the most noteworthy features of the most successful models. For example, the features taken from the left-hand for pronation, supination, and combined wrist motion were included in most of these top models. According to these results, left-handed supination frequency (*f*_3_) is evident in 33% of the models, while left-hand supination speed (*f*_1_) is present in 21% of the top linear regression models with low RMSE values.

While features with large effect sizes contributed to the regression models, it is important to control for model accuracy. As a result, the prediction accuracy of the best performing models among the 0.1% of all models (i.e., 262 models out of 262,125) was evaluated using Monte Carlo cross-validations using three different training versus test sample percentages: 90%, 75%, and 50% for training (versus 10%, 25%, and 50% for testing). The mean RMSE values for the trained models against the expected results were as follows: $\mathrm{mean}\left({\mathrm{RMSE}}_{90}\right)=1.37\pm 0.05,\;\mathrm{mean}\left({\mathrm{RMSE}}_{75}\right)=1.44\pm 0.06,\;\mathrm{mean}\left({\mathrm{RMSE}}_{50}\right)=1.60\pm 0.10$. Upon further examination of the UPDRS scores for the GP subgroup, it became apparent that it was a sum of four measurements on a five-point scale. In computing RMSE errors as a ratio of the maximum GP score, the following error percentages were obtained: 6.85%, 7.18%, and 7.98% with the three different training sets, respectively. Hypothetically, if a physician makes a one-point error in each of the four measurements in the UPDRS scores, the physician-based error is likely to be as high as 20%. In other words, the human error in GP scores may be much higher in comparison to the errors of our linear regression models. After examining our data, we found one UPDRS evaluation for which the two physicians disagreed, resulting in a four-point difference between their scores. For the rest of the sessions, however, there was a smaller UPDRS score difference between the two physicians ($\mathrm{mean}\left({\mathrm{dU}}_{\mathrm{GP}}\right)=1.06\pm 0.82$). Based on a percentage conversion of the total GP score, the percent deviation had a $\mathrm{mean}({\mathrm{dU}}_{\mathrm{GP}}^{\%})\;\mathrm{of}\;5.31\pm 4.10\%$, which was on par with the RMSE error levels.

When considering the co-occurrence of some of the $f_{1},\;f_{2}$ and $f_{3}$ features in multiple rows of the top 1% of the linear regression models in Figure 4, one wonders if these features might be linearly related, and may thus be able to be combined through principal component analysis (PCA).

## 4. Principal Component Analysis (PCA)

After PCA, we were able to make three observations. First, only 3 new components (i.e., new features) are enough to explain more than 97% of the data variance. When the individual feature contributions are calculated, it is seen that $f_{2}$ is the dominant feature followed by $f_{1}$, whereas $f_{3}$ has almost no contribution in all three components. Second, the pronation- and supination-related features are greater than the combined wrist feature. In a sense, this is an expected result since the wrist motion is the linear combination of pronation and supination. Third, the acceleration related features, namely $f_{2}$ related features, are the most prominent contributors of the new feature space. This is reported similarly in the previous correlation analyses.

As seen from Table 2, the $f_{1}$ and $f_{2}$ features are closely related and their combination in the first three principal components reflects 97% of the variance in the data. Accordingly, the 18 variables that represent the features in the linear regression models presented in Figure 4 can be reduced to form the three new features (i.e., the first, second, third components) derived from the PCA without losing accuracy to represent the entire feature space. However, the significance of *f*_3_ has been prominent in the models presented in Figure 4, even though the features related to *f*_3_ are represented with very small weights in the PCA. In other words, *f*_3_ is an important independent variable to predict GP, but it is not related to other features linearly, so it has little weight in the new features created by PCA. Hence, in the linear regression, we could keep all of the six features related to *f_3_* but replace the features constructed from *f*_1_ and *f*_2_ with the newly generated three features from PCA, to keep the linear regression’s predictive power high. With this reduction, the number of features in the linear regression would be reduced to nine.

## 5. Discussion

The present study provides evidence that GP and BA are correlated with the wrist supination and pronation speed, acceleration and frequency of the forearm. The findings demonstrated the potential for unilateral wrist movements to be used in the collection of information about gait and bulbar anomalies while patients are seated (without a need for patients to perform a sit-to-stand task). Such movements can be collected using unilateral wrist wearables, such as smartwatches.

The three P/S movement parameters assessed in this study showed that speed or feature f1 was significantly correlated with at least one or more of the five UPDRS motor scale subgroups (tremor, rigidity, bradykinesia, gait–postural instability, and bulbar anomalies). More importantly, the acceleration parameter of forearm supination was correlated with GP ability. Considering the bilateral correlation of forearm P/S acceleration with GP ability, the role of P/S as a proxy for GP ability was evident. The results of our analysis also indicate that P/S provides a better indication of GP when the non-dominant hand is used. This may be due to the specialization of the dominant hand/arm for tool manipulation; therefore, the dominant hand only supports a weak representation of global postural movements. On the other hand, the non-dominant hand may have maintained relatively stronger integrity with the higher-order centers of the GP control.

Gait and posture are traditionally considered to be the abilities that allow primates to stand on their lower limbs, and hence forearm muscles’ actions are excluded from the concepts or scoring systems that investigate gait and posture. In this context, clinical examinations including UPDRS part III for the motor functions of PD patients do not consider forearm pronation–supination to be a proxy for GP in PD patients, but rather bradykinesia. Evolution of locomotion in mammals, and especially adaptation processes in primates, provides evidence that forearm flexion/extension contributes to sustaining posture and gait stability [4,5,6,7,8,9,10,11,12,13,14,15,16,17,18]. The ability to maintain an erect posture during quadrupedal terrestrial locomotion has been linked to the ability to pronate the forearm [4,5,6,7,8,9,10,11,12,13,14,15,16,17]. By selectively retaining their supination ability, weightier mammals, such as those in the ursidae family (e.g., bears), may maintain forearm pronation better than lighter animals, with supination contributing to higher-order centers of gait and posture stability control [15]. Despite the emphasized morphological and evolutionary signs of a potential correlation between P/S motion data and GP ability, no investigations to date have assessed the potential contributions of P/S to GP ability. Accordingly, in the present study, we investigated the potential correlation between P/S and GP ability using different movement parameters such as speed, acceleration, and frequency. Five different components of motor functioning in PD patients were also assessed in subgroups established previously [23,24] using the UPDRS part III motor scaling system. With the combination of the five different components of UPDRS and the three movement parameters, we provided novel insights into potential correlations of P/S. In the present study, the UPDRS part III (motor scale) was used to score bilateral motor symptoms (ipsilateral and contralateral to the dominant symptom side). This bilateral extremity function scoring approach enabled us to have insights into the bilateral motor condition of patient motor symptoms and their correlations with GP ability.

In addition to GP correlations, our results further emphasized a correlation between P/S speed and BA-related UPDRS III in both forearms. It is worth noting that BA functions provide insight into the control of bulbar center CPGs [30,31]. The BA subscore of the UPDRS reflects dysphagia- and dysarthria-related dysfunctions, including swallowing and speech impairments, which are coordinated sequential movements. BA functions themselves are believed to be controlled by the CPGs [30,31], which is considered highly active and sophisticated in primates [32]. CPGs are proposed as key players in enabling bipedals to use quadrupedal coordination to control their gait [15,16]. It has also been shown that the upper limb demonstrates similarities with the hind limb only in the rhythmic movements [15,16,18,19]. Recent investigations also demonstrated the neuronal coupling of the upper and lower limbs [33]. The propriospinal neurons with their long axons are shown to be the link between the cervical and lumbar segments of the spinal cord [34,35,36,37]. The present study also supports the neural coupling of CPGs with the upper and lower limb movement, in the context of rhythmic upper limb movement (P/S) parameters to be used as a proxy to determine the BA and GP.

In addition to CPGs and spinal cord level coordination of upper and lower limbs, cortical level coordination cannot be excluded. Multi-array cortical recordings of the human premotor cortex demonstrated that the neurons in the hand knob area are involved in the movement of all four limbs being not specific to the hand [21]. Future studies are needed to investigate the spinal and/or cortical contributions to upper/lower limb coordination and their contributions to gait maintenance based on upper limb muscle movements.

While the UPDRS III scale is an observational assessment commonly used by neurologists to determine motor functioning in PD patients, its qualitative (mild, moderate, severe) components for scoring (0 to 4, 0 is none-normal, 4 is severe) decrease its objectivity. Given this, numerous motion capture systems have been proposed for the objective assessment of motor functioning in PD patients. Currently, wearable motion tracking systems for the full-body can provide gait and posture measurements. However, wearable systems for the forearm have been reported to produce high resolution data on tremor and bradykinesia, for example, but not for GP or BA. To the best of our knowledge, until now, no wearable systems have been assessed for correlations between GP and P/S, or GP outcomes from a single limb of a seated patient. Therefore, this study provides the first evidence of the correlation between P/S speed and GP in patients with PD. In the future, these findings could be used to develop applications for smart wearables that can predict G/P function without requiring patients to stand or walk. Therefore, it may be possible to remotely monitor GP stabilization abilities in Parkinson’s disease patients with low-cost, easy-to-use devices, including smartwatches.

UPDRS is a subjective scoring system that is based on physicians’ physical examination findings. Given this, we sought to minimize the role of subjective, individual physician judgment in traditional UPDRS III scoring by incorporating mean UPDRS scores from two neurologists. Additionally, bilateral UPDRS scoring was incorporated into our analyses as opposed to traditional/routine, unilateral UPDRS scoring. Our interpretations were contingent upon the physicians’ subjective UPDRS scores; in this context the results of the present study should also be validated with data from a wearable motion tracking system, which would provide objective GP scores in comparison to the physician-based UPDRS. This is a potential future study to compare quantified outputs of gait and posture with the objective features from our P/S system. The present study results are obtained in the OFF-state of the PD patients. The OFF-state is also common in patients who are under medication (between doses of medications and within an hour after taking oral medication) and understanding the symptoms of the OFF-state may help physicians optimize their medication dosages and timing. On the other hand, the outcome of the present study needs to be investigated among the ON-state patients in future studies. The patients in the present study are in PD H&Y states 2 or 3. As a consequence, the outcomes of the present study may not be applicable to more severe (H&Y > 3) states of the disease. Several factors, such as the age of onset of the disease, side affected at the time of onset of the disease, genetic factors, and gender, could influence the results of this study. However, large scale studies are still required in order to investigate the potential effects of these variables.

In the present study, to the best of our knowledge, we demonstrated for the first time whether the rhythmic pronation–supination movement data from a single wrist was correlated with gait/posture stability in PD patients in the context of the CPG integrity of upper and lower limbs and their contribution to maintaining the gait. Future studies are needed to reveal the neuronal networks that contribute to correlations between P/S and GP ability. This will have significant implications for the development of wrist wearables like smartwatches that have GP monitoring capabilities. These devices are critical for PD because they can be utilized to monitor gait/posture stability objectively and remotely in PD patients’ daily routine, even while users are seated. As a result of this capability, clinicians could efficiently monitor PD patients’ GP, as well as their T, B, and provide timely assistance to those who were experiencing GP dysfunction.

Overall, the present study demonstrated that P/S movement parameters are potentially helpful in predicting GP and BA states in patients with PD. In conclusion, the results of the present study support the prospect of using P/S speed data to understand G/P and BA with wrist wearables, allowing objective remote monitoring of these symptoms in the daily routine of PD patients. This may lead to remote monitoring, optimization of the treatment, and early prevention of gait disability-related falls.

## Figures and Tables

**Table 1:** Patients demographics of 17 male, 7 female patients with disease duration ± SD = 8.04 ± 3.88 years, mean age ± SD = 57.08 ± 8.91 years.

Age	Gender	Dominant Hand	Affected Side at Onset	P.D. Duration (year)	H&Y Stage
61	F	R	R	12	3
46	M	R	L	4	3
55	M	R	R	8	2
48	M	R	R	12	2
54	M	R	R	6	2
48	M	R	L	8	2
61	M	R	L	6	2
71	M	R	R	17	2
52	M	R	R	2	2
61	M	R	R	8	3
56	M	R	R	7	2
47	F	R	R	10	2
63	M	R	R	8	2
58	F	R	R	15	2
54	M	R	R	9	3
70	F	R	R	8	3
64	M	R	L	1	2
45	F	R	L	4	2
71	M	R	R	5	2
45	F	R	L	8	2
63	F	R	R	4	2
72	M	R	L	13	2
45	M	L	R	8	2
60	M	R	R	10	2

**Table 2:** Three components found by PCA and contribution of the components. $f_{1}$ and $f_{2}$ related features are the largest components whereas $f_{3}$ is always the smallest.

First Component (Covers 87.17%)		Second Component (Covers 9.01%)		Third Component (Covers 2.13%)	
f2_SUP_R	0.5090	f2_PRO_R	0.5835	f2_SUP_R	0.6509
f2_PRO_L	0.4509	f2_SUP_L	0.5583	f2_PRO_L	0.5245
f2_SUP_L	0.4441	f2_PRO_L	0.3377	f2_PRO_R	0.4362
f2_PRO_R	0.4267	f2_SUP_R	0.2974	f2_SUP_L	0.2566
f2_WRIST_R	0.2303	f2_WRIST_L	0.2218	f1_PRO_L	0.1435
f2_WRIST_L	0.2186	f2_WRIST_R	0.2049	f1_PRO_R	0.1080
f1_SUP_R	0.1148	f1_SUP_L	0.1176	f1_WRIST_L	0.0734
f1_WRIST_R	0.1066	f1_PRO_R	0.1094	f1_SUP_R	0.0580
f1_PRO_R	0.0977	f1_WRIST_L	0.0996	f2_WRIST_R	0.0483
f1_PRO_L	0.0890	f1_WRIST_R	0.0879	f2_WRIST_L	0.0322
f1_SUP_L	0.0884	f1_PRO_L	0.0812	f1_WRIST_R	0.0247
f1_WRIST_L	0.0880	f1_SUP_R	0.0651	f1_SUP_L	0.0110
f3_SUP_R	0.0013	f3_PRO_R	0.0015	f3_PRO_L	0.0016
f3_SUP_L	0.0011	f3_SUP_R	0.0010	f3_SUP_R	0.0015
f3_PRO_L	0.0011	f3_SUP_L	0.0009	f3_PRO_R	0.0008
f3_PRO_R	0.0010	f3_PRO_L	0.0004	f3_SUP_L	0.0002
f3_WRIST_R	0.0003	f3_WRIST_R	0.0003	f3_WRIST_L	0.0001
f3_WRIST_L	0.0003	f3_WRIST_L	0.0002	f3_WRIST_R	0.0001

## Data Availability

The datasets generated and/or analyzed in the current study are partially available (restrictions apply to the availability of these data due to Ethical issues and Ethical approval) from the corresponding author on reasonable request.

## Supplementary Materials

The following supporting information can be downloaded at: https://www.mdpi.com/article/10.3390/s22051827/s1. Figure S1: Pearson’s correlations between the extracted motion features from the right hand and the ipsilateral UPDRS scores from the right hand; Figure S2: Pearson’s correlations between the extracted motion features from the left hand and the ipsilateral UPDRS scores from the left hand; Figure S3: Pearson’s correlations between the extracted motion features from the right hand and UPDRS scores for the left hand; Figure S4: Pearson’s correlations between the extracted motion features from the left hand and UPDRS scores for the right hand; Figure S5: The data analysis algorithm chart, Video S1: 3D reconstruction of a patient performing P/S task that involves rapid, repetitive movements around the roll axis.
