# Supplementary material for: Hand Pronation–Supination Movement as a Proxy for Remotely Monitoring Gait and Posture Stability in Parkinson’s Disease
Source: Sensors (Basel). 2022 Feb 25;22(5):1827. doi: 10.3390/s22051827 (PMC8915024; doi:10.3390/s22051827)
Supplement: Supplementary file 1 [file sensors-22-01827-s001.zip › sensors-1486842-supplementary.pdf]

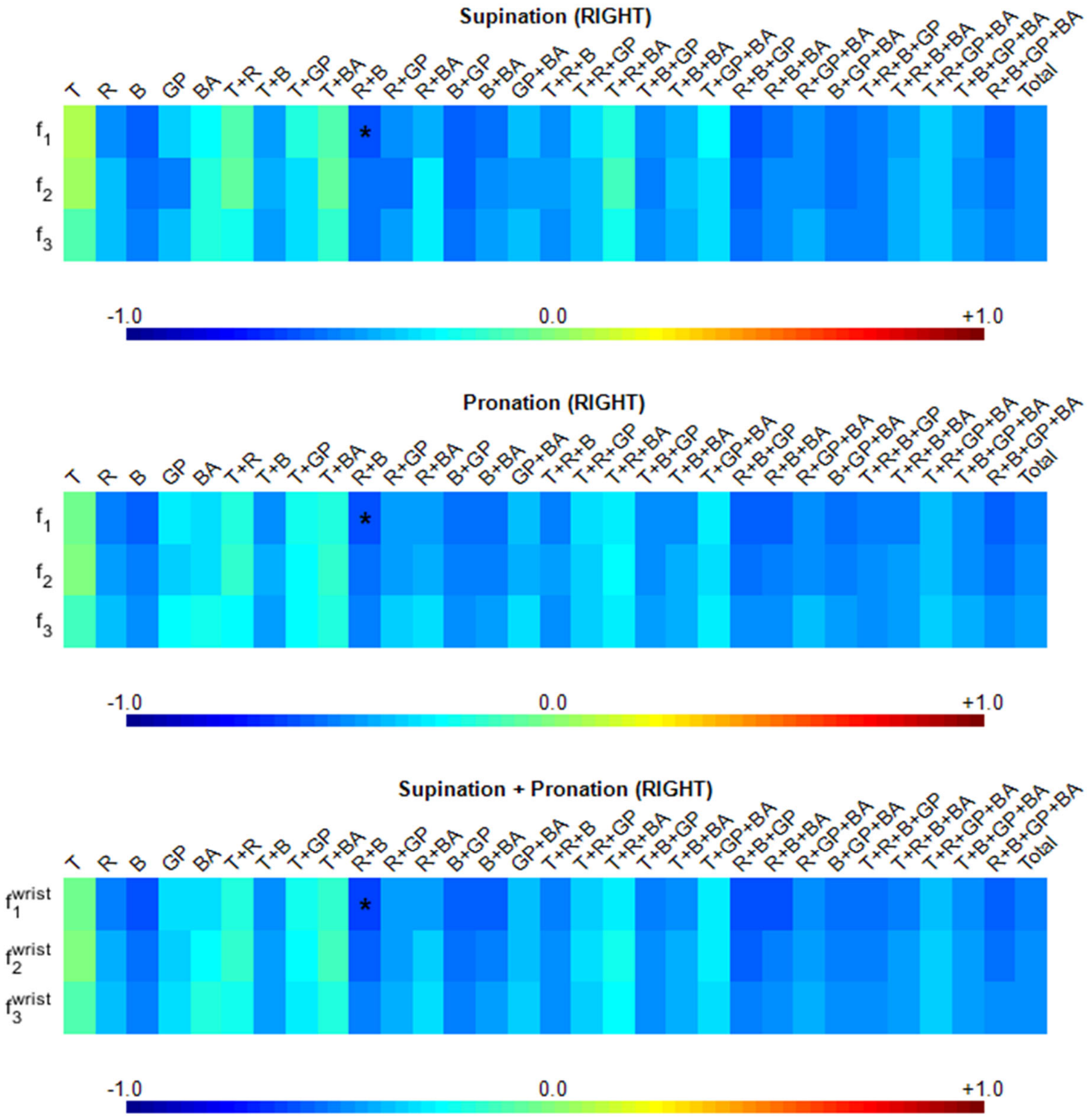

Figure S1. Pearson's correlations between the extracted motion features from the right hand and the ipsilateral UPDRS scores from the right hand (rows: features; columns: single and combined UPDRS scores).T=Tremor, R=Rigidity, B=Bradykinesia, GP=Gait and Postural Instability, BA=Bulbar Anomalies, * indicates strongest correlation; values above 0.2 and below -0.2 were significant at $p < 0.05$ level (Bonferroni corrected)

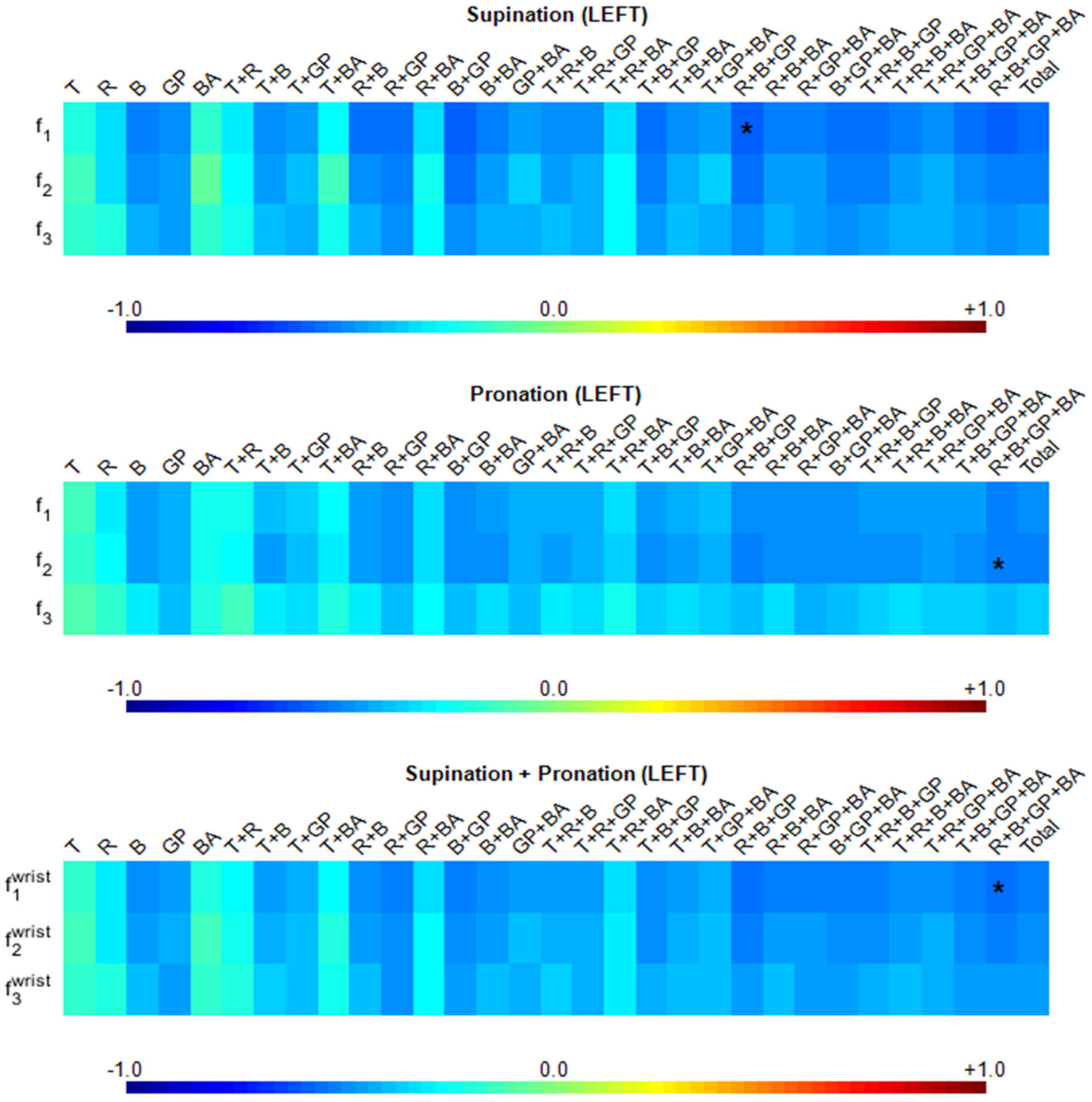

Figure S2. Pearson's correlations between the extracted motion features from the left hand and the ipsilateral UPDRS scores from the left hand (rows: features; columns: single and combined UPDRS scores).T=Tremor, R=Rigidity, B=Bradykinesia, GP=Gait and Postural Instability, BA=Bulbar Anomalies, * indicates strongest correlation; values above 0.2 and below -0.2 were significant at $p < 0.05$ level (Bonferroni corrected)

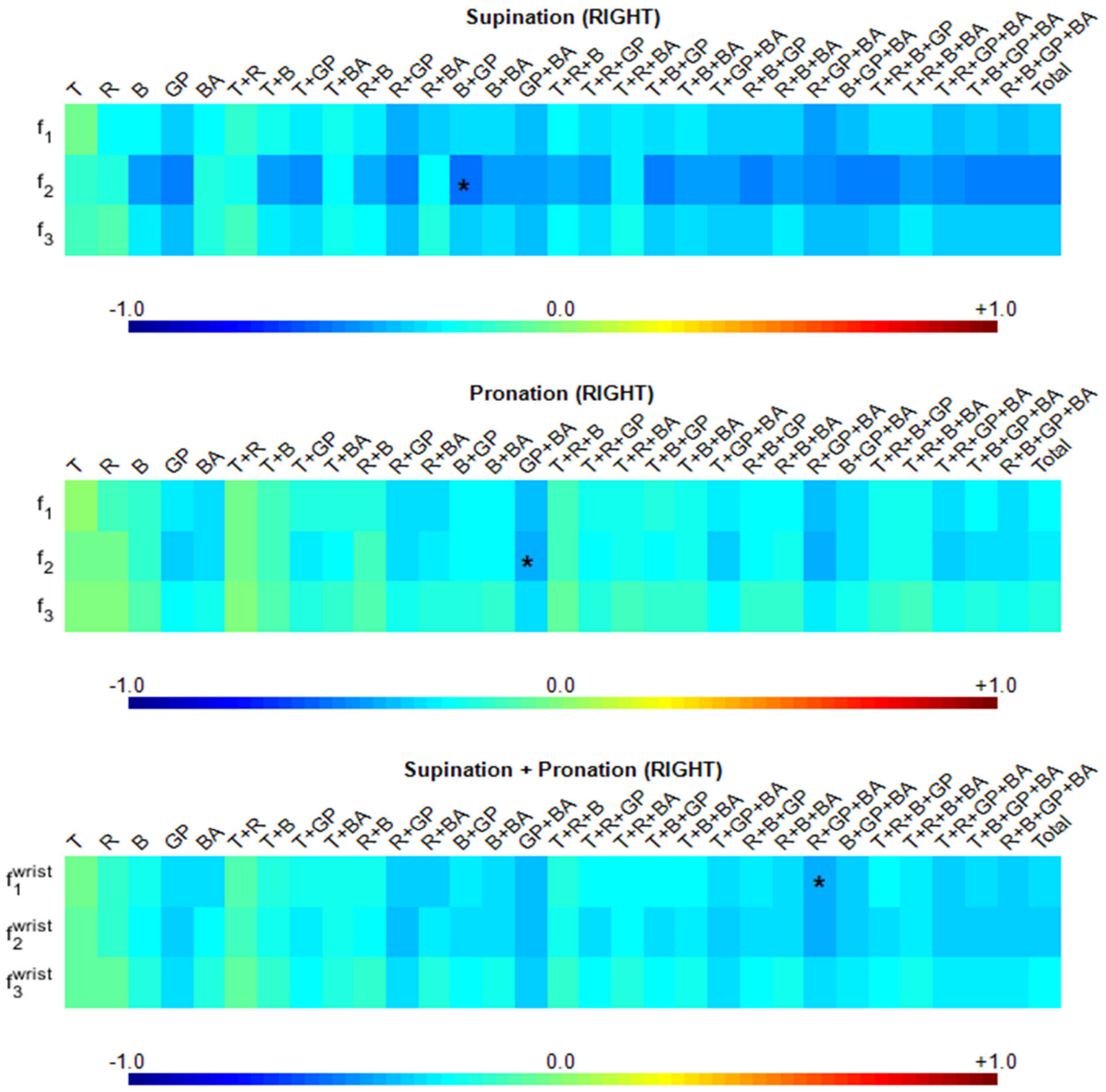

Figure S3. Pearson's correlations between the extracted motion features from the right hand and UPDRS scores for the left hand (rows: features; columns: single and combined UPDRS scores).T=Tremor, R=Rigidity, B=Bradykinesia, GP=Gait and Postural Instability, BA=Bulbar Anomalies, * indicates strongest correlation; values above 0.2 and below -0.2 were significant at $p < 0.05$ level (Bonferroni corrected)

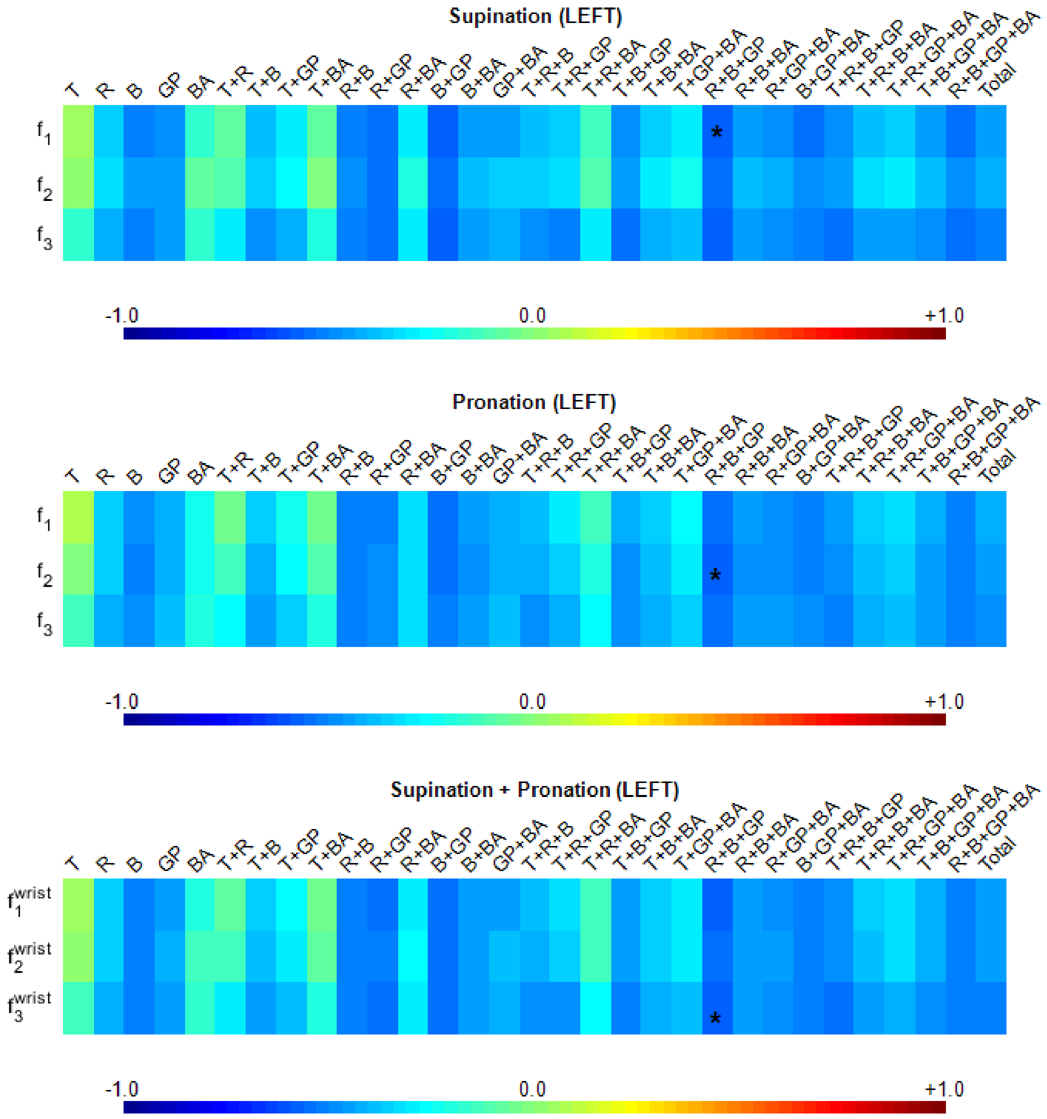

Figure S4. Pearson's correlations between the extracted motion features from the left hand and UPDRS scores for the right hand (rows: features; columns: single and combined UPDRS scores).T=Tremor, R=Rigidity, B=Bradykinesia, GP=Gait and Postural Instability, BA=Bulbar Anomalies, * indicates strongest correlation; values above 0.2 and below -0.2 were significant at $p < 0.05$ level (Bonferroni corrected)

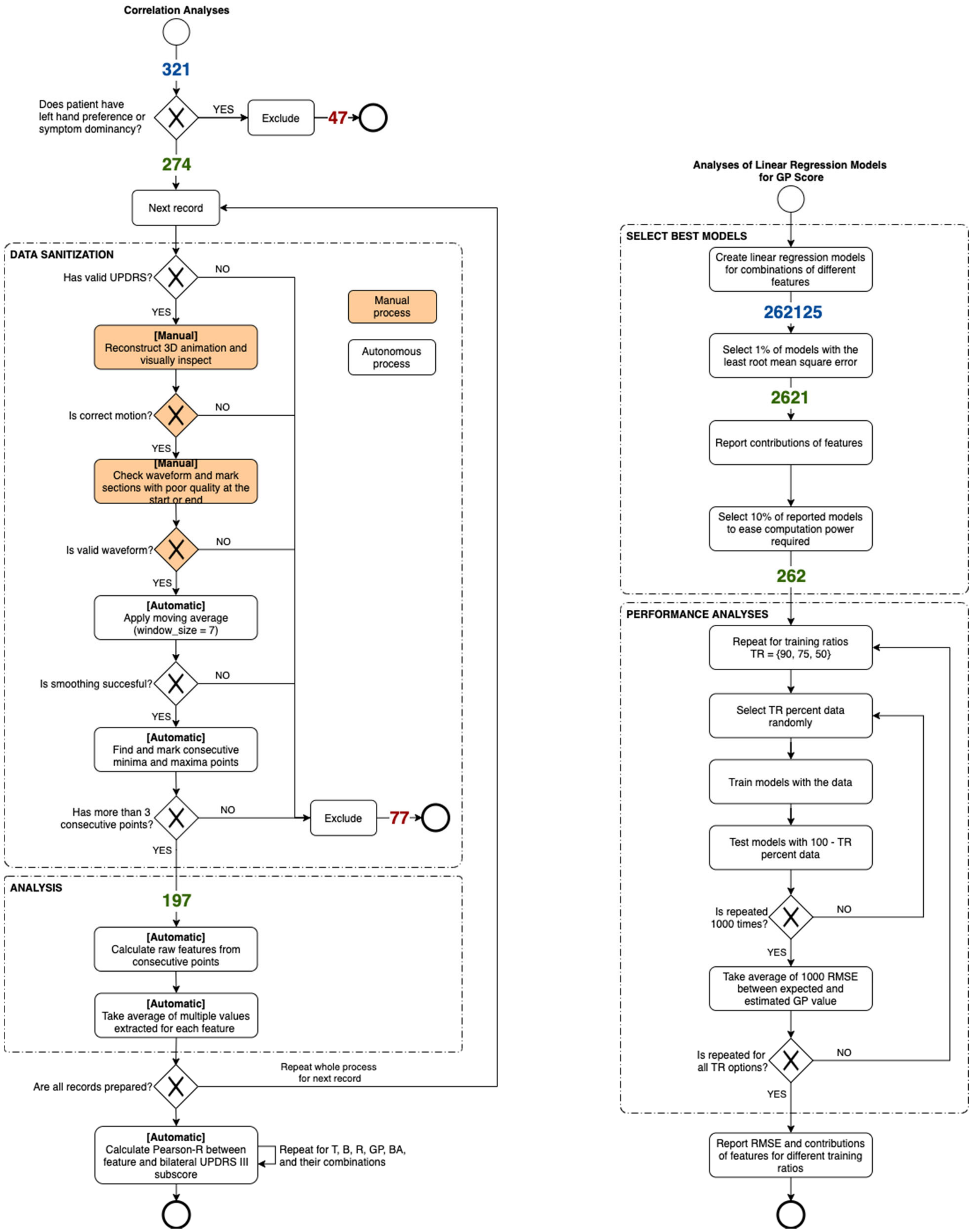

Figure S5. The data analysis algorithm chart
